# Balancing competing rationales in priority-setting in primary healthcare – an interview study on political governance

**DOI:** 10.1108/JHOM-10-2024-0438

**Published:** 2025-02-18

**Authors:** Suzana Holmér, Barbro Krevers, Kristin Thomas, Ann-Charlotte Nedlund

**Affiliations:** Department of Health, Medicine and Caring Sciences, Linköping University, Linköping, Sweden; Swedish National Centre for Priorities in Health, Department of Health, Medicine, and Caring Sciences, Linköping University, Linköping, Sweden

**Keywords:** Governance, Primary healthcare, Priority-setting, Rationales, Politicans

## Abstract

**Purpose:**

Publicly funded healthcare systems struggle to govern and determine how finite resources should be allocated in relation to political goals within a pre-determined budget. Primary healthcare (PHC) has a central multipurpose function, not least in terms of political strategies, but PHC governance is still largely underexplored. The aim is to explore how politicians responsible for making decisions pertaining to healthcare coverage navigate the governance of public PHC and disentangle it in the form of narratives based on different types of underlying rationales.

**Design/methodology/approach:**

Semi-structured interviews were conducted with 15 politicians from 3 Swedish regional healthcare authorities. The data were analysed abductively based on scientific, clinical and cultural rationales using thematic content analysis.

**Findings:**

Our study provides insights into how PHC’s multipurpose function implicates tensions between politicians' different responsibilities regarding healthcare coverage. It shows how politicians navigate various coexisting rationales, with some being more dominant than others and where tensions also exist between them. In this balancing act, they create narratives addressing different stakeholders and priority-setting dilemmas, reflecting the diverse rationales. Our study reveals that politicians play a crucial role in PHC governance and priorities, balancing rationales that might otherwise become overly dominant.

**Originality/value:**

This paper contributes new knowledge by displaying how politicians balance tensions within and between rationales through different narratives regarding goals/commissions, problematic situations and preferred solutions in PHC governance.

## Introduction

Publicly funded healthcare systems struggle to govern and determine how finite resources (i.e. money, professionals and time) should be allocated in relation to political goals, unstable health expenditures growth and citizens’ limitless healthcare needs due to advances in treatments and screening procedures (Seixas *et al.*, 2021; Kuhlmann *et al.*, 2024). This crucial task of deciding on what is covered (or not covered) is made at all levels in a healthcare system: within a pre-determined budget for each area of care, between areas of care, on a macro level between principal and contracted providers and even across sectors. This article focuses on primary healthcare (PHC), which has a central multipurpose function within a publicly funded healthcare system covering various areas and sectors.

PHC serves as a means of diminishing inequities in health by providing affordable and accessible care for the population (Starfield *et al.*, 2005; Van Lerbergerg, 2008; Rasanathan *et al.*, 2011). A review concludes that PHC is a useful strategy in reducing pressure on the overall health systems by acting as a gatekeeper to more expensive specialists (Starfield *et al.*, 2005), especially in the pursuit of cost-effective organisation (Starfield *et al.*, 2005; Van Weel and Kidd, 2018). PHC has gained extensive responsibilities and has high expectations of providing a wide range of care services to achieve political goals, such as availability, disease prevention and patient education (Van Weel and Kidd, 2018; Rasanathan *et al.*, 2011). Availability in terms of time and geography, as well as the organisational structure of PHC, is important to have a robust PHC (Starfield *et al.*, 2005; Van Weel and Kidd, 2018), and it has also been argued as vital for building and retaining the public’s trust, so they want to continue paying taxes to fund healthcare systems. However, studies have shown that the context in which healthcare professionals make priorities is changed by decisions and reforms made by politicians, regulators and policymakers (Arnell, 2011; Vengberg *et al.*, 2021). Consequently, in the everyday work of PHC healthcare professionals, deciding what is prioritised (and what is not) does not follow a “one fits all” solution, as priorities are adapted to fit the situation, patients' influx and previous decisions on healthcare coverage (Holmér *et al.*, 2023).

Despite PHC having a multipurpose function, not least in terms of political strategies, the governance of PHC is still largely underexplored. However, previous research on healthcare governance and resource allocation at large, including its priorities, suggests that “real-world” priorities in “the complex multi-layered governance context” (Kislov *et al.*, 2021) are often unformalised and implicit (Rooshenas *et al.*, 2015; Kislov *et al.*, 2021) and are usually based on historical decisions (Cromwell *et al*., 2015; Seixas *et al.*, 2021). Studies on priority setting have focused on enhancing the allocation process by developing and improving the formal framework of priority setting and its criteria and tools (Mitton, 2002; Williams *et al.*, 2011; Kislov *et al.*, 2021). A systematic review (Seixas *et al.*, 2021) revealed that a wide range of stakeholders were involved in formal priority-setting processes, including administrators, government officials and healthcare professionals. Politicians were involved, but their political interference was less prominent when frameworks were more robust. Previous studies show that governance arrangements demand a more dynamic relationship between politicians and healthcare professionals (Williams *et al.*, 2011; Garpenby and Nedlund, 2016; Fredriksson *et al.*, 2019). It has been argued that politicians depend on guidance from experts, such as medical or economic experts, when making priority and rationing decisions in healthcare (Rodwin, 2001). This could be due to the constant changes in structural conditions and the involvement of stakeholders with different responsibilities and specific interests (Denis *et al.*, 2001; Dickinson *et al.*, 2011). In several qualitative studies (Dickinson *et al.*, 2011; Smith *et al.*, 2014; Garpenby and Nedlund, 2016; Fredriksson *et al.*, 2019), politicians’ relational leadership is identified as a critical strategy used in navigating in this dynamic setting. Additionally, a case study (Gille *et al.*, 2020) highlights the importance of political leadership when establishing what to exclude and not include in publicly funded healthcare coverage. Political leadership has been highlighted as essential in retaining legitimacy in times of austerity; several qualitative studies show that processes and decisions need to be considered both fair and trustworthy among the actors internally involved in the process – e.g. health professionals, managers, politicians (Nedlund and Bareoe, 2014) and the public (Griffiths, 2002; Gibson *et al.*, 2005; Robinson *et al.*, 2012; Gille *et al.*, 2020).

The political nature of allocating and prioritising, especially in contexts where simple solutions are unavailable, involves stakeholders constructing situation-specific priority-setting narratives by discursively deploying tensions and contradictions between different rationales (Kislov *et al.*, 2021). Increasing budgetary pressures may have pushed politicians, regulators and policymakers to engage more explicitly in priority-setting procedures and healthcare coverage decisions. Although important research has investigated the implications of prioritisation, budget cuts, policy reforms and decisions regarding PHC and PHC professionals (Carlsen and Nordheim, 2005; Werntoft and Edberg, 2011; Vengberg *et al.*, 2021; Holmér *et al.*, 2023), less attention has been paid to the role of politicians in the governance of public PHC. The aim of this study is to explore how politicians responsible for making decisions pertaining to healthcare coverage navigate the governance of public PHC, given the constraints of finite resources and limitless healthcare needs. This will be disentangled in the form of narratives based on different types of underlying rationales.

### Theorising governance as narratives based on different underlying rationales

One way of understanding PHC governance is to adopt a critical interpretive policy perspective. Following the taxonomy offered by Nedlund and Nordh (2018), various competing underlying rationales are in play in governance and its practices. They explain that rationales entail assumptions, norms and values that can justify a course of action, such as in the case of governance and practice. Rationales guide what constitutes a problem, commonly by fronting the problem’s inherent solution. What are regarded as appropriate solutions following one rationale might be denoted as irrational, illogical and senseless when framed within another. Multiple rationales run parallel to each other, although some can be more dominating than others and occasionally totally overarching (Garpenby and Nedlund, 2016; Nedlund and Nordh, 2018). In priority-setting, politicians interweave various rationales to justify their focus on specific areas, programmes, projects or processes in priority setting (Klein, 2010; Garpenby and Nedlund, 2016; Kislov *et al.*, 2021).

In healthcare governance, stakeholders create narratives based on underlying rationales, where different features from particular contexts and arguments are brought to the fore (Lehoux *et al.*, 2010; Nedlund and Nordh, 2018; Garpenby and Nedlund, 2022). In a study on the influx of controversial health technologies, Lehoux *et al*. (2010) show that medical specialists combined arguments based on different rationales to create a purpose for why technologies should be used in which some assumptions *“[…]* *are explicit, while others remain tacit. Some are brought forward while others are omitted.”* A study by Garpenby and Nedlund (2022) on the interface between the patient and different actor groups in health care, including politicians, showed that the same actor group can connect to and display various rationales and can also shift narratives and arguments depending on the situational context (societal or individual and systemic or professional accounts). Following this theoretical reasoning, politicians create narratives in their governance of PHC and simultaneously employ different narratives based on different rationales.

In the present study, drawing from Nedlund and Nordh’s (2018) and Lehoux *et al*.’s (2010) taxonomy, we use three rationales – scientific, clinical and cultural – as an analytical framework in PHC governance analysis.

Scientific rationale acknowledges positivistic science and technocratic reasoning and emphasises the importance of facts and evidence in the discovery of “truth” (Lehoux *et al.*, 2010; Nedlund and Nordh, 2018). Knowledge achieved through scientific studies such as clinical trials and randomised controlled studies is held in high regard. Scientific rationale focuses on aspects related to evidence, such as collecting enough data to determine the effectiveness of a therapy and ensuring studies are carried out to a high standard (Lehoux *et al.*, 2010). Policies within this rationale are regarded as value-free. Scientific rationale is also driven by ideas such as economic reasoning in terms of cost-effectiveness, streamlining and optimisation. Furthermore, technical/technocratic solutions or better scientific knowledge may solve social and political problems (Nedlund and Nordh, 2018). This knowledge is held by individuals or institutions claiming expert knowledge who also significantly influence the cause of action (Nedlund and Nordh, 2018).

A clinical rationale emphasises clinical relevancy, feasibility, standardisation of care and practices and clinical know-how. Lehoux *et al*. (2010) describe that even effective treatments/innovations supported by robust scientific evidence might not be used for patients for various reasons. For instance, the side effects could restrict patients from participating in their everyday lives, or there could be issues including such treatments in care units, as units might lack necessary medical specialists or require extensive training for professionals to learn the skillset necessary (Lehoux *et al.*, 2010). Additionally, standardisation of clinical practices or guidelines is essential to ensure quality of care. Nedlund and Nordh (2018) do not distinguish between scientific and clinical rationales. Still, they describe (referring to Stone, 1986) how clinical reasoning is a way of examining individuals, comparing fixed characteristics and making determinations – commonly based on clinical decision-making about who is entitled to get services. As such, clinical reasoning functions as a means of gatekeeping. Hence, a clinical rationale is concerned with assessing and classifying potential patients based on health states. In contrast to scientific rationale, healthcare professionals' judgement and proficiency (their “clinical know-how”) are acquired through clinical experience and practice.

A cultural rationale encompasses the beliefs, norms, values, desirability and expectations in society and confers messages about the capacity of society to solve collective problems (Nedlund and Nordh, 2018) and is, therefore, considered in healthcare governance. Cultural values, such as solidarity, ideas about contributing to common welfare by paying taxes and responsibility for how taxes are allocated, can also be included within a cultural rationale (Nedlund and Nordh, 2018). Lehoux *et al*. (2010) refer to this rationale as “social”, explaining that even scientifically proven effective and feasible innovations may not be adopted due to cultural norms, ideologies, stigmas or religious connotations. They explain that politicians might hesitate to provide treatments and screening because such decisions could prove controversial. The cultural rationale behind potential stigmas related to how specific health diseases/treatments, as well as historical instances of healthcare institutions' abuse of power, influence who should get what services (Lehoux *et al.*, 2010; Nedlund and Nordh, 2018). Aspects that consider inequity, such as whether a service is available to everyone or only for those who can afford it, are also prominent. Commonly, the role of politicians in PHC governance has been presented as being the citizens’ representatives, a narrative based on a cultural rationale.

### The Swedish healthcare context

This present study is conducted in Sweden, where taxes fund public healthcare. In the Swedish healthcare system, 21 regional healthcare authorities are the main providers responsible for governing the provision of healthcare for their citizens at hospitals and PHCs. A total of 290 municipalities also provide PHC in inpatient settings, such as in nursing homes and through home care. Regions and municipalities have locally elected politicians and independent powers of taxation, which grants them considerable autonomy and empowers regional politicians to govern healthcare in alignment with what they see as best for their citizens. For PHC, regional politicians formulate explicit goals and requirements that care units must fulfil. Politicians at the national level have the power to exercise and govern healthcare through legislation, regulation, financial incentives, agreements and agenda-setting.

The Swedish healthcare system aims to achieve good and equitable health in the population by prioritising publicly financed resources. Sweden is one of the only a few countries (Sabik and Lie, 2008) that have explicit ethical prioritisation principles on how to allocate resources and has stipulated this in law since 1997 (Government bill, 1996; Health and Medical Services Act, 2017). According to Swedish law, the *human dignity principle* is an overriding principle, stating that all people have equal value and the same right to care, regardless of personal characteristics or their function within society. The *need solidarity principle* implies that those in greater need – those with more severe diseases and worse quality of life – should be granted a more significant portion of common healthcare resources. Finally, *the cost-effectiveness principle* (lexically ordered) stresses the need for a reasonable relationship between a treatment’s cost and its effects. These principles serve as a basis for how common resources should be distributed. They are meant to guide professionals, managers and national, regional and local politicians in prioritising and rationing decisions.

## Method

We applied a qualitative explorative design. Data are based on individual interviews with regional, locally elected politicians responsible for providing healthcare services. These politicians were members of regional political boards or committees directly influencing PHC resource allocation, such as implementing new digital entryways for patients seeking PHC or changing the financial compensations. To increase variation in data, purposive sampling in the recruitment of politicians was performed by recruiting participants from different political parties in three regional healthcare authorities, with variations in size (250,000–450,000 registered inhabitants), organisational structures and available healthcare services. All participants were recruited through their public email addresses that were obtained from their political parties or the region’s official webpage. The email contained an information letter and contact information. Two reminder emails were sent, and one follow-up phone call was conducted one week after the last email reminder. In total, 15 politicians (10 men and 5 women) were interviewed from September 2022 to January 2023.

A semi-structured topic guide with open-ended questions covering areas such as PHC assignment, health coverage, political control and the influence of political decision-making on healthcare practice through resource allocation and re-allocation was designed. Interviews were conducted online (14) and face-to-face (1). Each interview lasted between 40 and 85 min and was recorded and transcribed verbatim. The transcribed text was analysed using abductive thematic content analysis in accordance with Ritchie *et al*.’s (2003) “framework approach”. The research team includes a doctoral student (first author) and three senior researchers with extensive experience in qualitative methods, healthcare and policy research.

The first author (SH) became familiarised with the data by reading the transcripts several times. Text relevant to the study’s aim was highlighted and initially coded into scientific, clinical or cultural rationales (Lehoux *et al.*, 2010; Nedlund and Nordh, 2018). Within each rationale, three themes were inductively identified: goals/commissions, problematic situations and preferred solutions. From the first draft, written by SH, tensions between the narratives and within the rationales were identified and discussed with AN. The analysis and the manuscript text were revisited and developed in an iterative process with all authors to ensure the consistency and trustworthiness of the analysis. The participants responsible for the quotes in “Findings” have been pseudonymised (P1–P15) to protect confidentiality. According to Swedish legislation (Ethical Review Act, 2003), ethical approval is not needed for ethical human research, as the study did not include any intervention or sensitive data. The 2014 Declaration of Helsinki guided the study’s ethics (General Assembly of the World Medical Association, 2014). Before the interviews, all politicians received written information about the research and their rights, stipulating that their participation was voluntary and that they were free to withdraw at any time. They were assured of pseudonymisation. Participants verbally consented to participate before starting the interviews.

## Findings

The findings follow the theoretical framework, addressing scientific, clinical and cultural rationales. Within each rationale, three themes emerged (Table 1): (1) goals and commissions consist of politicians' views or understanding of what PHCs or politicians must provide for the healthcare system or society and why; (2) problematic situations consist of situations that politicians identify as problematic in their role governing PHC and (3) preferred solutions consist of ideas, perspectives or solutions needed to ease some of the problematic situations related to PHC governance. The three themes are presented as narratives based on each rationale.

**Table 1:** Overview of rationales and themes in politician’s narratives of primary healthcare governance

Themes	Goals and commissions	Problematic situations	Preferred solutions
Rationales			
Scientific	Being the healthcare frontline – PHC* prevents the use of more expensive specialist care and has a legal obligation to coordinate/integrate healthcare between healthcare authorities	Limited finances create challenges to compete for staff against other regions Medical experts' words carry a lot of weight but might have a narrow focus. Compensating wrong things could lead to crowding out of patients	Streamlining processes, implementing technical advances and law changes. Ideas hold by knowledgeable people. Law changes by national-level politicians. Financial incentives can motivate desired actions
Clinical	Being the healthcare frontline – PHC* prevents burdening higher healthcare levels. Politicians provide goals, guides, and aims for care units to fulfil	Lack of overview of PHC units creates difficulties in governing. Limited staff resources and organisational structures promote care at the wrong healthcare level. National-level politics affect the PHC* units processes	Improving structures, processes and management could increase the impact of governance. Feedback from professionals is needed to shape realistic compensation models and avoid displacing patients. Unspecified state grants and long-term solutions
Cultural	Being the healthcare frontline – PHC* prevents citizens from getting sick. Political mission includes responsibility for all citizens. Dialogues with professionals and citizens	Officials gain power if dialogues between politicians and professionals/citizens are limited. Equality aspects could be lost with certain solutions. Conflict between allocating resources vs preventive care. Social consequences. Lack of people who want to settle in rural areas	Enact political initiatives to impose change and secure resources. Collaboration with other authorities. Politicians’ opinion on how PHC* practices are organised. Strengthen citizens’ rights. Support others’ prioritisation decisions

**Note(s):** *PHC-Primary healthcare

### Primary healthcare governance narratives based on scientific rationale

Politicians described PHC as playing a central role in healthcare and referred to laws that obligate PHC to coordinate patient care with the municipality and maintain patient continuity. They also argued that, from an economic perspective, it is better for patients first to visit PHC since it prevents the use of much more expensive specialist care (hospital care). Politicians viewed healthcare professionals as experts in maintaining the central role of PHC (gatekeeper to the rest of healthcare) because of their knowledge regarding when referrals to other healthcare specialists are necessary.

… they [healthcare officials and the regional board] are pretty much in agreement that primary healthcare is the first line of care, it’s a relatively cheap form of care, and it should be easy to get in touch with –P3

… but we still have the responsibility to make sure the money is sufficient … to ensure that we’ll achieve the results we have promised … –P13

The governance of PHC was described as being managed through reimbursement models by financially compensating for specific measures or results that were of interest. Politicians highlighted boundaries in their ability to govern, stipulated either by national-level politicians, the region’s political and organisational structure or the politicians' power to adjust reimbursement models. For instance, politicians emphasised that financial compensation was applied to motivate PHC organisations to work towards their (the politicians') goals for PHC and achieve good quality of care. Nationally set goals and financial compensation affect regional PHC governance. For instance, national directions were often understood as *“not freely chosen work”* (P10) – whether or not they were stipulated by law – and politicians acknowledged it changed regional goals, especially if the national directions/goal fulfilments were combined with financial compensation. PHC governance was tightly entwined with politicians' ability to adjust their region’s reimbursement models. Politicians described not having the power to make demands or withhold financial compensation for those PHC practices that did not fulfil official requirements. In contrast, other politicians expressed that they could *“rule with both a stick and a carrot”* (P14) by not reimbursing unfulfilled goals or adding monetary compensation for goals that are of specific interest to them.

… Well, then we put money toward it and decided … that the care units may not be reimbursed for any number of listed patients with a physician [in PHC], and that's our way of reducing the number of listed patients per physician –P14

In this narrative, any issues with PHC governance are referred to as “challenges”. For instance, good accessibility rates for PHC were challenged by politicians' lack of freedom to employ personnel or compete for staff against other regions by offering higher wages because they lack financial resources. Meanwhile, solutions were attributed to freeing up time, which could compensate for staffing issues or poor PHC accessibility by streamlining administration, implementing new technical advancements and changing laws. For instance, instead of sending summons digitally, ordinary mail could save time.

Other time-saving solutions included allowing patients to book their own appointments digitally, contact their PHC centre through chat functions and allow PHC operations to offer *“net-based visits”* (P7) through video. Additionally, national-level politicians could, by changing the law, contribute to more effective use of PHC professionals time. One politician explained how the existing law allowed general physicians to recover for 48 h after weekend duties. However, some physicians use this recovery time to work in other regions or private alternatives, while the ordinary region’s PHC operations remain understaffed.

… which means that when physicians go on call on a weekend … they get a lot of free time, and the idea with that [free time] is for them to recover … but it doesn't work that way … then they go out and work somewhere else, in another region –P6

Individuals who were viewed as knowledgeable “experts” had a strong influence on long-term political decisions. These individuals were described as having a good reputation, being respected in their field of work, having had a long, successful career or having a wealth of personal knowledge. Politicians problematised how transferring decision-making to individuals seen as “experts” could lead to a narrowed focus. Referring to their experiences governing during the COVID-19 pandemic, politicians stated that they trusted their infection control physician’s advice on how to proceed with the vaccination regime for the region. However, they acknowledged that the expert focused solely on reducing the spread of infection.

… there’s always a political dimension, and I think it can be difficult: that politically we should have, maybe, been a little more involved and made decisions … although … it is difficult. Because I very much trust the professionals who give us advice, or who say ‘let’s do it this way’ –P3

… and we end up in this, it's a medical assessment, and no politician can do that -P9

In this narrative, financial compensations mandated by national-level politicians are described favourably. For instance, the measure of the national waiting-time guarantee, which guarantees citizens a PHC appointment within a set timeframe, was emphasised as a crucial, well-designed indicator because it triggers multiple actions and leads to evaluation. However, politicians problematised the waiting-time guarantee against prioritising resources according to the need and solidarity principles and acknowledged there is a conflict, as patients with less severe healthcare needs are not necessarily those waiting the longest. Moreover, it is easy for care units to *“trick”* (P9) measurements to get good results (and financial compensation) by changing organisational structures, patient flow or processes. One politician explained how some PHC care units only made appointments one day at a time; when the daily ratio is exhausted, patients are asked to call back the following day. Patients would be registered as having had no waiting time, *“but I would say they had waited for two days”* (P8). Politicians stated that it is an *“important, democratic question”* (P2) of what is measured and compensated for in PHC, as “*we might not measure what we need to measure”* (P2).

### Primary healthcare governance narratives based on clinical rationale

In the clinical narrative, PHC’s role is described from a medical and clinical perspective; PHC plays a central role in providing medical assistance to patients, which helps to avoid placing unnecessary, non-medically motivated burdens on units at higher healthcare levels. Moreover, politicians explained that because large volumes of care throughout life will be managed in PHC, PHC plays a coordinating role for patients.

… but I imagine that it will be the big trawler that goes across the sea and pulls up as much as possible. And that is the basic idea; that primary care should sort and triage –P15

According to politicians, a lack of resources decreases PHC accessibility and affects how patients seek care, which obstructs goal fulfilment. Politicians argued that patients sought care at higher healthcare levels instead of waiting a little longer. However, they described that healthcare professionals also contributed to poor use of resources by over-referring patients to higher healthcare levels, such as to the emergency room (ER). Explanations for healthcare professionals' behaviour were that they did not know the patient, had limited work experience in general at that specific healthcare centre or had a large turnover of rental staff. On the contrary, even experienced healthcare professionals could refer patients to the ER for convenience instead of handling them themselves. According to politicians, healthcare professionals could understand the impact patients' healthcare needs would have on their own workload. Organisational structures could also contribute to patients being helped at the wrong healthcare levels. One politician explained that after-hours PHC is non-strategically placed next door to the ER and that some patients seek care at the ER since it takes them the same amount of time to get to the hospital. Additionally, healthcare professionals' work coordinating patients’ healthcare was complicated by the statutory confidentiality, which exists between municipalities and regions.

… yes, it could be out of convenience, too. You think ‘that //if I start this, I understand that it will be a lot’ … And then I might as well [refer patients to the ER] … –P3

… but it is difficult to work operationally, so to speak, in cooperation with, for example, student health … which should really be the first contact for our children and young people, but then … there are still two different authorities, two different legislations which makes a sort of wall between them [regional and municipalities authorities] –P9

In the clinical narrative, the impact of governance and resource use could be increased with improvements to clinical operations, for instance, by changing structures, processes or managers. Politicians explained that PHC struggled with accessibility through the telephone, as patients could not reach their providers when they called. They suggested that clinical operations could increase accessibility by offering daily drop-in times without telephone pre-assessment, as patients could be attended to directly. Politicians believed that if patients could seek care whenever they wanted, some would wait longer at home and perhaps even recover independently, not needing medical assistance at all. Moreover, having “*good managers*” (P7) and *“strong leaders”* (P8) was highlighted as necessary and even crucial for successful governance. Politicians exemplified how some PHC operations finally fulfilled their political goals and succeeded in establishing good practices by changing managers.

… how important it is to have a good manager … Because if you didn't have that presence in management on the business floor, it really didn't work when there was such an overload in the organisation –P15

In the clinical narrative, difficulties in governance relate to a lack of political oversight regarding the organisation and its operational focus. Therefore, healthcare professionals' feedback on the impact of political decisions on clinical operations was described as essential. Politicians emphasised the necessity of feedback to avoid neglecting patients who needed more time getting undressed, examined or treated to shape realistic compensation models. According to the politicians, the need for feedback became especially prominent during COVID-19; since professionals were focused on *“managing another day”* (P4), politicians' insights into operations were minimal. Additionally, politicians were concerned with how local- and national-level political decisions impact clinical operations. For instance, state grants meant to reduce waiting times for patients' initial psychiatric appointments yielded impressive statistics for first-time visits. Still, patients had to wait up to two years before treatment could start. According to politicians, these state grants would affect their region’s resource allocation and clinical operations, as long-term financing ended up being a regional responsibility due to the grant’s short validity time. These state grants were often not applied for due to their overly detailed focus, which, according to politicians, would not work in practice. However, state grants with less specificity (like those used during COVID-19) would be more beneficial for regions and could avoid detail management at the local level.

… so, large and wide is fine, but for God's sake, don't make sure we have more details in this governance [state grants] … because it … doesn't work in practice when it looks different, and there are different needs … it could be that this region has already worked very well [and cannot be granted the money] –P2

### Primary healthcare governance narratives based on cultural rationale

In this narrative, PHC plays a central role in relation to the idea that the political mission is to be responsible for all citizens. Politicians emphasised that their responsibility and political mission is to govern while keeping all citizens in mind, not only patients. For instance, politicians argued that PHC is important in preventing citizens from getting sick by offering preventive measures. According to politicians, citizens also have a personal responsibility to engage in self-care to prevent sickness.

I think that primary care also plays a fairly large role in the preventive and outreach work of the healthcare system when it comes to public health and public health issues –P5

Politicians have a civic starting point for ideas or solutions that could be beneficial. For instance, they expressed concern for how comorbid patients are treated, as they tend to be sent around in the healthcare system as *“ping-pong balls”* (P4). Moreover, *“from a citizen’s perspective”* (P2), PHC must be easily accessible, as a simple phone call could suffice to achieve a sense of security for the caller. By adapting opening hours to its community and enabling access to care through digital means for faraway citizens, accessibility can be achieved. Politicians included inclusivity as one accessibility aspect. They argued that information should be accessible in various forms rather than only online or via text, as not all citizens are technically knowledgeable or literate. Moreover, politicians emphasised that PHC facilities should be included in municipal city planning when building new neighbourhoods to be where citizens are and to fulfil their central role. They highlighted the importance of continuity through a *“stable”* healthcare contact that knows patients and their families.

… yes, but it is how we are citizens of a society, and one part is about the municipality that has municipal healthcare, and for the regions, it’s about healthcare [regions are responsible for healthcare in municipalities], and citizens are caught in between –P13

In the cultural narrative, human interaction is a vital form of PHC governance, and according to politicians, transparency in governance is achieved through dialogues with citizens, municipalities, members of the same and other parties. However, based on politicians' experiences governing during the COVID-19 pandemic, the strained situation forced regions, municipalities and politicians across all political parties to cooperate and *“just solve things*” (P1), and more extensive political discussions were put on hold. Even though all political meetings were held digitally, politicians emphasised they could not fully compensate for the lack of face-to-face conversations, as people were not as talkative *“through Zoom as in real life”* (P1) and meeting spots that facilitated spontaneous dialogues vanished when everybody worked remotely. Politicians emphasised that as the dialogues stopped, the power to govern was transferred to the officials. They further elaborated that even though officials informed them of a citizens' perspective about how things were in healthcare, they had difficulty grasping the whole picture, as they understood the information to be filtered.

… as a politician, going around the care units and asking for things, that is, quite literally … setting up meetings with people and asking questions, it’s an underestimated form of governance … and perhaps it is a means of control that we underestimate … because it's not like this formally, // … there are no protocols –P4

According to the politicians, national-level governance is welcomed if it strengthens citizens’ rights and promotes health equity without transferring power to the state. The law stipulating a patient’s right to choose a healthcare provider was exemplified as a good example of national-level governance. Other *“reasonable”* (P5) national-level decisions are those that promote equity between the 21 healthcare regions. In contrast, politicians emphasised risks, given national-level politicians’ 2023 election promises to nationalise healthcare. According to politicians, knowledge about local society, experiences, traditions and local collaborations and citizen assembly work is essential to adapt healthcare to citizens’ needs, but with nationalised healthcare, everybody would be required to do things the same way, and health equity could be thwarted.

… there’s not the same tradition, and then to demand that everyone should sort of do this in the same way, which easily happens when the state is giving an assignment, I think there is a risk, a big risk with that –P5

In this narrative, political initiatives can help PHC uphold its goal and include other community areas that might extend outside the borders of healthcare. For instance, politicians described that to secure and increase the long-term foundation of healthcare professionals, some parts of the medical education programme were expanded to their region, as *“those who move to university usually do not move back home*” (P1). Other initiatives included facilitating entertainment services or helping certain stores open up to attract people to settle in smaller cities. However, they emphasised that such political initiatives do not always work due to fear of consequences: for the citizens or for themselves as politicians. For instance, there will probably not be any change in the law allowing general physicians to recover for 48 h after weekend duties because national-level politicians fear the social consequences that will follow a physician’s strike, *“and we all know who will get the sympathy”* *(*P6). Additionally, politicians state that they must support those who have made tough choices when setting priorities, even though citizens might express discontent and not re-elect them.

… and that's where politics has to be fully supported … there have actually been times when the patients don't get what they want, complaint to politicians who then do not support [the managers], but back down, saying ‘and yes, but of course, this patient should have the right to this and that’ and that's not okay –P10

In the cultural narrative, all issues related to prioritisation concern aspects of health equity. Politicians highlight that not everybody can enjoy PHC services, as complicated processes might require active patients to complete all steps. They acknowledge that in their role as politicians, it might be necessary to express opinions on how PHC clinical operations organise themselves to make healthcare services accessible for all citizens. Moreover, politicians acknowledge that offering preventive care – when resources should be prioritised according to healthcare needs, creates an issue, as healthy citizens compete with already-ill patients, what is to be considered illness or disease is not always clear, and the threshold determining when healthcare is needed is uncertain.

## Discussion

This study contributes to the knowledge about governance of public PHC by demonstrating how politicians lean on various rationales (scientific, clinical and cultural rationales) in PHC governance. It sheds light on nuances in politicians’ governance and reveals how politicians, due to budgetary pressure, are pushed to govern PHC with strategies not only based on historical decisions. Moreover, it illustrates tensions within and between different rationales when prioritising resources according to healthcare needs.

Our research illuminates the political role of navigating diverse narratives and, consequently, different strategies for handling similar problematic situations. Within all narratives, a lack of personnel was described as the main reason PHC has difficulties upholding its goal/commission of being the frontline of healthcare services. However, within each rationale, attention is directed to different solutions, such as technical advances (scientific), organisational improvements (clinical) and expanding medical education (cultural). Hence, these preferred solutions require politicians to use different strategies, as the solutions are feasible within different time spans and require different levels of involvement, dialogues and agreements with various stakeholders or authorities to succeed. Other researchers (Denis *et al.*, 2001) have pointed out that political change is driven by cyclical or sporadic patterns. Our research complements these findings by showing that navigating narratives and strategies also follows political temporality and cycles. It shows that, at times, it might be more tactical for politicians to use different narratives or to not introduce preferred solutions that could entail major consequences when it is time to determine PHC budgets, as external and internal opinions carry weight.

Tension exists between narratives and within and between rationales. For instance, within the scientific rationale, there is tension regarding the use of financial incentives combined with certain indicators to motivate care units to align with important political goals. Assessments might be essential when concluding that political decisions are carried out and in retaining the public’s trust in welfare (Gille *et al.*, 2020). On the other hand, only depending on what aspects of care are measured and financially compensated (Vengberg *et al*., 2021) could crowd out greater healthcare needs, potentially undermining the achievement of equitable care and the population’s good health and eventually harming public trust. However, the narrative based on the clinical rationale suggests that politicians balance these tensions through feedback from healthcare professionals by shaping financial incentives that mirror everyday work to prevent systematic crowding out of patients who require more time to be examined or treated. The clinical and cultural rationales exemplify tensions between the two solutions. For instance, the clinical rationale focuses on change and improvements in organisational processes and structures, while the cultural rationale focuses on who benefits from the organisational improvements in terms of fulfilling political goals and adhering to the budget, compromising equality. Previous studies have shown that the operational aspects of care units matter when it comes to who eventually gets access to care (Werntoft and Edberg, 2011; Holmér *et al.*, 2023). Our finding implies that politicians must voice their concerns regarding solutions driven by care units affecting how patient groups are prioritised (or not prioritised). Politicians also need to monitor how such solutions affect the pre-determined budget.

Politicians' power to govern healthcare coverage can be limited, even with various governance strategies available. Relational leadership has been emphasised as a key role in priority-setting (Reelder *et al.*, 2006; Williams *et al.*, 2011), as politicians might need to use different strategies to address the power dynamic within healthcare (Denis *et al.*, 2001; Garpenby and Nedlund, 2016). Our findings complement these assumptions and show how politicians use different narratives, addressing different stakeholders, medical experts (scientific), healthcare professionals/managers (clinical) and the public (cultural) to keep their decision-making power. These narratives are useful for addressing various priority-setting dilemmas. Politicians might end up as bystanders due to leaning on experts (Rodwin, 2001); however, as shown in our study, this may also be the case in situations where feedback from care units and dialogues with citizens are lacking. Hence, relational leadership seems important in political governance so that decisions are based on the best available knowledge, create a good working situation for healthcare professionals and are best for long-term healthcare resourcing.

A noticeable finding is that there seem to be tensions between various political responsibilities: being accountable for all citizens and maintaining their trust in welfare by allocating common resources to those with greater healthcare needs and promoting preventive measures for healthy citizens – who compete for the same finite resources as healthcare measures for patients – all while staying within the pre-determined budget. Our research suggests that politicians attempt to navigate these equal responsibilities via different narratives in a political temporality.

### Methodological considerations

In qualitative research, purposive sampling is an appropriate technique when recruiting participants with different experiences governing various political organisations, ensuring data richness (Patton, 2014). The findings' credibility is enhanced by the thorough description of the participants, the topic guide and the data collection and analysis processes (Patton, 2014; Korstjens and Moser, 2018). We consider that our research group’s methodological and subject competence and the constant movement between data, analysis and writing strengthen the study’s credibility (Ritchie *et al.*, 2003). The results from our qualitative methods can be used in future studies to develop quantitative studies to capture a larger sample. We consider the findings to be transferable to other democratic healthcare systems that offer public PHC. Moreover, we acknowledge that the rationales might be weighted differently in different healthcare systems.

## Conclusion

Our study provides insights into how PHC’s multipurpose function implicates tensions between politicians' different responsibilities regarding healthcare coverage, such as prioritising and distributing common resources and maintaining the public’s and staff’s trust. Our study shows how politicians navigate various coexisting rationals with some being more dominant than others and where tensions also exist between them. In this balancing act, they create narratives addressing different stakeholders and priority-setting dilemmas, reflecting the diverse rationales. It displays the complexity of the political role, how different narratives are used to address priority-setting dilemmas and how governance must be adjusted to the context, especially in PHC. Our study reveals that politicians play a crucial role in PHC governance and priorities, balancing rationales that might otherwise become overly dominant.
